# Complete Genome Sequence and Methylome Analysis of Micrococcus luteus SA211, a Halophilic, Lithium-Tolerant Actinobacterium from Argentina

**DOI:** 10.1128/MRA.01557-18

**Published:** 2019-01-24

**Authors:** F. L. Martínez, B. P. Anton, P. DasSarma, V. Rajal, V. Irazusta, R. J. Roberts, S. DasSarma

**Affiliations:** a Instituto de Investigaciones para la Industria Química, Consejo Nacional de Investigaciones Científicas y Técnicas, Universidad Nacional de Salta, Salta, Argentina; b Institute of Marine and Environmental Technology, Department of Microbiology and Immunology, University of Maryland School of Medicine, Baltimore, Maryland, USA; c New England Biolabs, Ipswich, Massachusetts, USA; University of Delaware

## Abstract

Micrococcus luteus has been found in a wide range of habitats. We report the complete genome sequence and methylome analysis of strain SA211 isolated from a hypersaline, lithium-rich, high-altitude salt flat in Argentina with single-molecule real-time sequencing.

## ANNOUNCEMENT

Micrococcus luteus strains have been isolated from a wide range of sources, including the air (1), soils (2), and humans (3). We isolated an extremophilic strain of M. luteus designated SA211 from soil of the hypersaline Salar del Hombre Muerto in northwestern Argentina that can grow in media containing high concentrations of salts (up to 4 M NaCl), including lithium (up to 30 g/liter in defined liquid media) (4). Genome sequencing was performed to establish methods for its use in biotechnological applications.

Genomic DNA was isolated from a culture of M. luteus SA211 grown to late exponential phase at 30°C and 200 rpm in modified nutrient broth (MNB) (4) using the following procedure. Cells were harvested by centrifugation at 10,000 rpm for 10 min (Eppendorf Centrifuge, 5415R) and lysed by adding 800 μl of lysozyme (10 mg/ml) in buffer (75 mM NaCl, 25 mM EDTA, 20 mM Tris, pH 7.5) and incubation for 2 h at 37°C (5). Next, 80 μl of 10% SDS and 20 μl of proteinase K (20 mg/ml) were added, and the mixture was incubated for 2 h at 55°C. Then, 335 μl of 5 M NaCl and 700 μl of chloroform:isoamyl alcohol (24:1) were added, and the mixture was incubated for 30 min at room temperature. It was then centrifuged at 12,000 rpm for 10 min, and the aqueous phase was recovered. DNA was precipitated by the addition of sodium acetate (3.0 M, pH 5.2) and isopropanol (0.65 volumes) and pelleted by centrifugation (12,000 rpm, 10 min) (6). The pellet was washed with ethanol and dried, and the DNA was resuspended in TE buffer with pH 8 and stored at −20°C.

Sequencing was performed with the PacBio RS II platform. A SMRTbell sequencing library was prepared from genomic DNA randomly sheared to 20 kb with a Megaruptor (Diagenode, Denville, NJ) and size selected (6 to 50 kb) with a BluePippin system (Sage Science, Beverly, MA). The library was sequenced with one single-molecule real-time (SMRT) cell with C4-P6 chemistry and 360 min collection time. Sequencing reads (37,323 total, with mean subread length 12,515 bp) were assembled *de novo* with RS_HGAP_Assembly.3 (7) in the SMRT analysis 2.3.0 environment with the default settings. Error correction and closure were performed with RS_BridgeMapper.1, and methylation patterns were determined with RS_Modification_and_Motif_Analysis.1, both also within the SMRT analysis environment and with default settings. Annotation was performed in house with GeneMark (8), tRNAscan (9), and the NCBI Prokaryotic Genome Annotation Pipeline (PGAP) (10).

The 2,483,045-bp genome is GC-rich (73%) and consists of a single, circular chromosome. It codes for 2,161 predicted proteins, with a median pI value of 5.6 and a mode pI value of 4.8, which indicates an acidic proteome characteristic of halophiles (11). It also codes for 48 tRNAs and possesses 2 operons with 5S, 16S, and 23S rRNAs.

The methylated DNA motifs and the methyltransferases (MTases) predicted to be responsible for each are shown in Table 1, with locations of methylated bases on the top strand (A or C) and bottom strand (T or G) underlined. FMM_11335 is the most likely open reading frame (ORF) responsible for AGCCCA because it resembles other Type IIG MTases methylating a 3′ terminal A residue; however, another ORF, FMM_01825, remains a possible, but less likely, candidate. The motif CTCGAG manifested as 2 related sites, CTCGAGNR (40.1%) and CTCGAGVNB(N_11_)B (15.1%); the exact methylated base is unknown.

**TABLE 1:** Methylated motifs in M. luteus SA211

Motif	Modification	% motifs detected	Predicted MTase locus
GCA(N_7_)RTGT	m6A	100	FMM_08165
CAGATG	m6A	100	FMM_10000
AGCCCA	m6A	99.9	FMM_11335
CTCGAG	Probable m5C	15 to 40	FMM_08635

### Data availability.

The M. luteus SA211 genome sequence has been deposited in GenBank under the accession number CP033200. Raw data are available in the NCBI Sequence Read Archive under the accession number SRP166874.
